# Exercise and Atrial Fibrillation: Some Good News and Some Bad News

**DOI:** 10.22086/gmj.v0i0.1401

**Published:** 2018-11-08

**Authors:** Alireza Sepehri Shamloo, Arash Arya, Nikolaos Dagres, Gerhard Hindricks

**Affiliations:** Department of Electrophysiology, Heart Center Leipzig at University of Leipzig

**Keywords:** Atrial fibrillation, Exercise, Primary Prevention, Aging

## Abstract

Atrial fibrillation (AF) is considered as the most common sustained arrhythmia in adults, whose incidence rate is on the rise due to the increase in the mean age of the global population. In recent years, many efforts have been made to identify effective factors in the incidence of AF to prevent them and thereby reduce the consequences of AF. Physical activity is one of the topics that attracted much attention in the last two decades. According to some findings, extreme and prolonged exercise itself can be considered as a risk factor for the onset of AF; however, other studies have shown that exercise can be regarded a protective factor against AF in the general population. The present study reviews the findings of studies on the relationship between AF and exercise and discusses possible mechanisms for this relationship. Additionally, we present some recommendations for researchers and physicians about exercise management in association with AF prevention.

## Introduction

Atrial fibrillation (AF) is the most commonly reported sustained arrhythmia in adults, with an estimated prevalence of 1-2% in the general population [1,2]. Due to the aging population of the world and the increasing prevalence of risk factors associated with AF, we are expected to witness an AF epidemic in the coming decades [3]. Therefore, the identification of possible mechanisms and triggers of AF, especially the detection of preventable agents, and appropriate preventive programs can play a significant role in promoting community health and reducing the costs associated with disease management [4]. The general belief is that regular exercise can improve cardiovascular health and reduce cardiac events [1,5]. In fact, physical activity is considered as an appropriate measure in preventing the risk of heart diseases by controlling weight gain and blood pressure as well as enhancing cardiometabolic efficacy [3,6]. Indeed, studies have shown that regular exercise leads to a reduction of 30-50% in all-cause mortality [7]. However, recent evidence suggests that extreme and prolonged exercise itself can be considered as a risk factor for the onset of AF [8].

On the other hand, many studies have shown that exercise can be a protective factor for AF in the general population [9]. Over the past two decades, more than 40 different studies have attempted to provide evidence of how physical exercise is linked to the risk of developing AF [5,7,10]. Most of these studies are design-oriented in two overall categories: 1) studies that have taken place in the general population with the purpose of showing the effects of physical activity or inactivity on the risk of AF; and 2) studies that have been performed in athletes, sometimes compared with the control group, aimed at revealing the effects of intense exercise on the risk of AF. In this paper, we review the findings of a variety of studies in this area and discuss possible mechanisms regarding the relationship between exercise and AF. Lastly, we present some recommendations for further research in this field.

## Studies in the General Population

More than ten population-based studies have been conducted so far, to investigate the impact of physical activity on the risk of AF incidence [5,10]. In the majority of these studies, evidence suggests that mild to moderate physical activity can reduce the risk of AF in the community, whereas only few studies provide evidence of the ineffectiveness of regular exercise in attenuating the risk of AF [7,11,12]. Results of the Aizer et al. study in a population of more than 16,000 people showed that habitual vigorous exercise for 5-7 days per week in people under 50 years of age increases the risk of AF, while there is no association between the incidence of AF and exercise in less time duration and at ages older than 50 years [11]. Similarly, in another study in Sweden, higher level of exercise in men of lower age was associated with an increased risk of AF, while in older men, cycling/walking was associated with reduced AF risk [13]. Two studies from Norway and Finland showed that physical activity in women [12] and men [14] was not associated with a decrease in AF incidence.

In contrast with the studies showing a non-significant or reversed relationship between physical activity and AF burden, other studies have suggested that daily walking or cycling can reduce the risk of AF [5,7,15]. In a study in Sweden, it was found that moderate physical activity could be effective in reducing the risk of AF in women (related risk: 0.85 for ≥4 h/week vs. <1 h/week) [16]. In the Cardiovascular Health Study, it was concluded that the incidence of AF in older adults with more regular physical is around a half lower than those with less physical activity [17]. In recent years, two Asian studies were also conducted to complete the Asian section of the puzzle of the relationship between exercise and AF. In both of these studies, which were collectively performed in a population of about 375,000 people, it was shown that moderate physical activity plays a protective role against the onset of AF [18,19].

Recently, some other evidences have approved what was shown by most of the population-based studies [7,20,21]. The findings of these studies, which include three cohort studies by 2018, suggest that better cardiorespiratory fitness is associated with a 30-60% lower risk of AF during follow-up of 5-19 years [7,20]. Another study also found that any 1- metabolic equivalent (MET) increase in cardiorespiratory fitness could reduce the risk of AF by 7% (1 MET = a whole-body oxygen consumption of 3.5 mL O2/kg/min) [21]. Also, in other studies in the United States and Norway, results are also in favor of the effectiveness of exercise in reducing the incidence of AF [1,5,22,23]. Although many population-based studies have been carried out so far, we still cannot see a very precise model for estimating the effect of minimal physical activity, depending on its condition and type, in relation to AF risk; however, the beneficial effects of exercise are unobtainable on the overall pattern of heart disease risk. By examining the sum of evidence, it is possible to claim that mild to moderate physical activity in the general population can be matched with the expected decrease in the risk of AF incidence, whereas physical inactivity or heavy exercise, especially in young men, increases the risk of AF.

## Endurance Exercise

As described above, according to population-based studies, vigorous physical activity, especially in young men, could be accompanied by an increased risk of AF. Nevertheless, in vitro studies on animal models, clinical trials with heavy exercise induction, case-control studies on AF patients, and cohort research on professional athletes have reinforced the hypothesis of the risk factor nature of intense exercise for AF in recent years. The first evidence related to the risk factor nature of extreme exercise for AF was reported in the late 1990s [24], while in recent years there have been other studies that uncovered a U-Shape and/or J-Shape relationship between exercise and risk of AF [6,25]. Studies have reported that athletes are about 2-4 times more likely to have AF than the “normal” population [26,27]. Other investigations in marathon runners, skiers, and cyclists, especially in long-distance cross-country skiing and cycling, have underlined that there is a higher incidence of AF than the general population; and every 10 years of participation in professional sports results in a 20% increase in the AF risk [11,28,29]. It was also found that moderate to severe activity in excess of 2000 hours during lifetime was significantly associated with a higher incidence of AF (odds ratio=3.88; 95% confidence interval= 1.55-9.73) [30]. Another study on 52.755 long-distance cross-country skiers reported that the incidence of AF was significantly higher among those who had reached the finish line faster and completed more matches [31].

A meta-analysis exhibited that the AF risk rate in athletes was 5.3 times more than in non-athletes [32]. This odds ratio of around five is impressive when compared with the odds ratio of about 1.5 for the proven relationship between high blood pressure and increased risk of AF [5]. Other meta-analyses have also been published in recent years, which reported that the risk of AF in relation to the extent of exercise follows a J-shaped pattern [22]. However, among studies conducted in athletes, two studies with a sample size of less than 100 people have shown that endurance exercise is not necessarily associated with structural changes in the heart and thus the risk of AF [33,34]. These two studies may not question the overall orientation of the other studies for the direct association between intense exercise and increased risk of AF, but they can provide new hypotheses about the mechanisms that cause structural changes in the heart, and development of AF associated with extreme exercise. For example, can any kind of intense exercise be associated with increased risk for AF development or is this association restricted only to some particular types of severe exercise? Can the duration of exercise and its continuity be recognized as a risk factor in the incidence of AF?

## Possible Mechanisms

To investigate the possible mechanisms in relation to the effect of exercise on preventing or increasing the risk of AF, it is better to consider the mechanisms by assuming the J-Shape association of exercise with AF in two general categories: 1) the mechanisms of AF induction through exercise, and 2) protective mechanisms of exercise against AF.

### Mechanisms Explaining an Increased Risk for AF

To better understand the mechanisms that are associated with AF induction through exercise, we categorized them in two groups: 1) for athletes with physical endurance activity, and 2) mechanisms associated with the lack of physical activities (sedentary lifestyle). Although there is still no precise mechanism for increasing AF among athletes with severe physical activity, some important pathways for the underlying pathophysiology have been proposed [35]. Ectopic triggers (originated from the pulmonary veins), modulators as well as altered atrial substrate are three potential mediators suggested for the athlete’s atrial arrhythmogenesis [36,37]. More precisely, elevated autonomic activity, bilateral atrial dilatation, atrial fibrosis (possibly due to increased aldosterone following exercise), abrupt shifts between vagal dominance and sympathetic drive, increased atrial premature beats, changes in the ion channels of the pacemaker cells, and cell junction loss and collagen accumulation (possibly due to increased vascular thickness, similar to what happened for blood pressure) are among the suggested mechanisms for increasing the risk of AF following endurance sports [10,38-41]. On the other hand, the lack of physical activity also increases systemic inflammation and oxidative stress; inflammation can induce atrial remodeling and eventually the incidence of AF [7,10]. Autonomic dysfunction and sympathetic tone enhancement are other results of sedentary lifestyle that can increase afterdepolarization-dependent triggered activity and thereby increase the risk of AF [10,16].

### Mechanisms Explaining a Decreased Risk for AF

There is still insufficient information available on the protective mechanisms of exercise against AF [5]. However, increased physical activity and better cardiorespiratory fitness may contribute to reducing the risk of AF by helping to prevent other AF-related illnesses such as obesity (by decreasing visceral fat), hypertension, diabetes (by improving glycemic control), and obstructive sleep apnea [42-47]. Further, improving systolic and diastolic function, reducing arterial stiffness, lower sympathetic drive, and making positive changes in the structure of the heart (such as decreasing the left atrial size) are among the other possible mechanisms in relation to the effect of physical activity on reducing the risk of AF.

## What to Do Next

### Suggestions for Practice

Based on existing evidence, mild to moderate-intensity physical activity for 150 to 200 minutes per week (about half an hour per day), or aerobic exercise for 90-150 minutes per week (about 15-20 minutes per day), or achievement of cardiorespiratory fitness over 8 seems to be associated with a lower risk of AF [9]. However, it is strongly recommended to determine the desired duration and intensity of exercise in coordination with the medical practitioner [23], especially in individuals with other AF risk factors.

### Suggestions for Further Research

Perhaps the most significant limitation of current studies is the presence of heterogeneity in the methodologies used to measure the level of physical activity associated with decreasing or increasing the risk of AF. On the other hand, the criteria used to detect AF do not fit in many studies, and this makes it hard to draw a comprehensive conclusion. In relation to measurements of activity level, there have been hitherto several methods, including self-reported data (based on qualitative variables such as low, moderate, severe, and very severe), daily exercise time report in hour, cardiorespiratory fitness status and calories intake measurement. Self-reporting of daily activities of participants in studies can create a report-bias, resulting in errors in the conclusion, which is suggested to be replaced in subsequent studies by other methods. Therefore, it seems that conducting further investigations, with a higher focus on more precise methods for measuring activity and AF, can provide a better evidence basis for clinical decision-making. Also, while many studies have been conducted to uncover the mechanisms of AF induction by heavy exercise, the protective mechanisms of exercise against AF have not yet been well understood, and further studies are needed in this area.

## Conclusions

According to published results, it seems that the level of physical activity and risk of AF have a nonlinear relationship. Regular “mild to moderate exercise” may have to be considered as an appropriate strategy to reduce the risk of AF in the general population. Moreover, avoiding a sedentary lifestyle or very heavy exercise may be also ways to prevent the onset of AF, especially in young men. Lastly, besides the exact potential mechanisms, one of the questions that remains partially unanswered is the effect of different types of sports, their frequencies and durations on AF incidence. So, further studies are needed in order to gain a better understanding of the association between sports with different properties and risk of AF.

## Conflict of Interests

None
